# The Experiences of Outreach Support Staff Working with People with Mild Intellectual Disabilities during Different Stages of the COVID-19 Pandemic in the Netherlands: A Qualitative Study

**DOI:** 10.3390/ijerph20021515

**Published:** 2023-01-13

**Authors:** Laura Vromans, Maria C. den Boer, Noud Frielink, Petri J. C. M. Embregts

**Affiliations:** Tranzo, Tilburg School of Social and Behavioural Sciences, Tilburg University, 5037 AB Tilburg, The Netherlands

**Keywords:** COVID-19, pandemic, intellectual disability, outreach support staff

## Abstract

The COVID-19 pandemic profoundly impacted the work of professionals who support people with intellectual disabilities. This study aimed to explore the experiences of outreach support staff supporting people with mild intellectual disabilities in the Netherlands during different phases of the pandemic between March 2020 and May 2021. Overall, seven outreach support staff from three intellectual disability services participated in this qualitative study. Using semi-structured interviews, participants were interviewed on three occasions between December 2020 and May 2021. A thematic analytical framework was used to analyze the interviews. Four overarching themes could be distinguished based on the data: (1) balancing between one’s professional and personal life; (2) vaccination as both a stress reducer and a source of agitation; (3) service users: vulnerable versus resilient; and (4) contact with colleagues and service users. These themes provided valuable insights into the experiences of outreach support staff during different phases of the pandemic, both in the enduring impact of the pandemic and its measures on support staff, as well as in terms of how the pandemic and its preventive measures impacted their profession.

## 1. Introduction

The coronavirus disease 2019 (COVID-19) was declared a pandemic by the World Health Organization in March 2020 [1]. Over the course of the pandemic, governments across the globe implemented a series of preventive measures to minimize the risk of infection, including the closure of schools, libraries, and other public spaces, and advising the public to stay at home, practice social distancing, and reduce their use of public transport [1]. While the pandemic had a deleterious impact upon the physical, mental, and/or social functioning of everyone, the consequences were particularly severe for vulnerable people, including people with intellectual disabilities and their support systems, which primarily comprise family members and care professionals [2,3]. Indeed, attempting to cope with the consequences of the pandemic had a profound emotional, cognitive, practical, and—in the case of their formal network—professional impact upon these support systems [2,4,5,6]. For example, care professionals reported that they were fearful of getting infected and worried that they had unknowingly worked with service users with intellectual disabilities who had COVID-19 without wearing adequate protective equipment. This ultimately induced uncertainty and anxiety over infecting both themselves and their family and friends [4,6]. Moreover, on an emotional level, care professionals experienced feelings of frustration, disappointment, and responsibility, which, in turn, could lead to a sense of feeling overwhelmed by the emotions resulting from the implications of the pandemic [4,5,7]. This is evidenced by the fact that care professionals working with service users with intellectual disabilities reported higher levels of distress during the initial stage of the COVID-19 pandemic compared to the period immediately prior to the pandemic [6].

Most of the studies examining care professionals working with service users with intellectual disabilities during the pandemic have focused on the experiences of direct support staff working within residential care facilities [4,5,8]. However, in most western countries, a large part of the population of people with intellectual disabilities receive outreach support. Whereas direct support staff who work in residential care facilities need to comply with the rules and regulations of their facility, outreach support staff visit service users in their private homes. According to Courtenay [9], service users who live more independently experienced particular difficulties in understanding the importance of following the preventive measures related to the COVID-19 pandemic. As a consequence, there may be considerable variation in the way that this specific group complies with preventive measures, which, in turn, may impact upon the outreach support staff who visit them.

Although most of the preventive measures associated with COVID-19 were eased after the initial stage of the pandemic in the spring of 2020, various restrictions remained in place for a significant period of time and, indeed, in numerous countries new preventive measures were, and still are, implemented [10]. A recent longitudinal study by Flynn et al. [11] showed that many people with intellectual disabilities who receive support at home from outreach support staff were not receiving the same level of support during the timeframe of their study (i.e., March 2020–September 2021) as compared to pre-pandemic. This loss of support from outreach support staff during the COVID-19 pandemic may not only have impacted upon service users, but also upon outreach support staff themselves. To investigate the actual impact on support staff, the present study aimed to explore the experiences of outreach support staff supporting people with mild intellectual disabilities during different phases of the COVID-19 pandemic in the Netherlands between March 2020 and May 2021.

## 2. Materials and Methods

### 2.1. Participants

Overall, seven outreach support staff members (hereafter referred to as support staff) from three intellectual disability services participated in this study. The support staff (six women) had a mean age of 38.9 years (SD = 6.5, range: 32–50) and, on average, they had worked with service users with mild intellectual disabilities for 12.4 years (SD = 4.3, range: 6–20). Although most of the support staff solely worked with adults with mild intellectual disabilities who independently lived in the community, two of the support staff also supported children within special needs education; during the analysis of the data, we exclusively focused on the experiences with adult service users.

### 2.2. Design and Context

A qualitative research design was adopted to longitudinally explore the experiences of support staff working with service users who independently lived in the community over the course of a specific period of the COVID-19 pandemic in the Netherlands (i.e., March 2020–May 2021). Within this specific period of time, using semi-structured interviews, the participants were interviewed on three separate occasions between December 2020 and May 2021. The first round of interviews took place in December 2020, when a partial lockdown was in force. At that time, the Dutch government had introduced measures on the number of daily visitors one could have (e.g., a maximum of two visitors a day at home), the size of groups that could congregate at one time, alongside implementing closures of restaurants and cafés, and requiring the public to wear masks on public transport and inside certain places. The second round of interviews took place in the first two weeks of January 2021. At that time, a strict lockdown was in place, which included, among other measures, the closure of schools, indoor sporting venues, and all non-essential shops. Notably, at the beginning of January, support staff were invited to receive their first COVID-19 vaccination [12]. Between 23 January and 28 April 2021, an evening curfew was in force. From 28 April onwards, society began to gradually reopen as the infection rate decreased and the vaccination programme progressed. The third and final round of interviews took place during this period (i.e., April–May 2021).

### 2.3. Procedures

After receiving approval from the Ethics Review Board of Tilburg University (RP149), the intellectual disability services affiliated with the Academic Collaborative Center Living with an intellectual disability at Tilburg University were invited to take part in the study. This invitation resulted in three intellectual disability services expressing their interest in participating. After being informed of the aim and nature of the study, the managers of these three services subsequently recruited eligible support staff by inviting them to take part via email or telephone. Upon support staff expressing interest in participating in the study, an information letter and informed consent form were then sent via email by the first author, and a first appointment was made to conduct an interview. All participants provided written informed consent.

All of the semi-structured interviews were conducted by the first author. Each interview began with the same open-ended question: How are you doing at this stage of the pandemic? The use of multiple open-ended questions like this allowed for a dialog to be established with support staff. An interview guide was used to ensure that all the required topics were discussed, while, simultaneously, remaining open to other relevant topics that might be raised by the support staff. In all rounds of interviews, support staff were asked about their experiences during the current stage of the pandemic, which involved discussions around comparisons and differences between their current experiences and earlier stages of the pandemic, as well as their fears and worries. During the first round of interviews, support staff were also asked to reflect on their experiences during the first stage of the pandemic. A similar interview guide was used during all rounds of interviews; albeit this was supplemented with topics deemed to be relevant to the current stage of the pandemic (e.g., the vaccination program and the implementation of new measures during the second and third round of interviews, respectively). During the subsequent round of interviews, the researchers’ interpretation of the former interviews was checked with the participants. The interview guides are available as an online supplementary file.

Due to the COVID-19 measures, the interviews were conducted using a video conferencing tool (i.e., Microsoft Teams); one participant preferred to be interviewed by phone. The interviews were recorded using the record function within Microsoft Teams, or, in the case of the telephone interviews, a voice recorder, and transcribed verbatim. The interviews lasted on average 29 min (range: 12–45).

### 2.4. Data Analysis

The interview transcripts were analyzed using the six-phase process associated with a thematic analysis framework [13]. After each round of interviews, the transcripts were first read in detail by the first and second authors in order to familiarize themselves with the interviews. Then, they coded phrases that appeared to be relevant to the study in 20% of the interviews. This was separately executed so that the inter-rater reliability could be calculated: an 84% level of agreement was reached between both authors. Any disagreements were discussed with the two other authors until a consensus was reached. The remaining eighty percent of the interviews were coded by the first author. Third, all codes were categorized into potential themes by the first author, before subsequently being critically reviewed by all authors. The codes that were unrelated to the aims and objectives of the study, such as statements concerning the support of children with mild intellectual disabilities, were discarded. Fourth, all authors examined the themes for internal homogeneity, external homogeneity, coherence, and relevance. Fifth, the different themes were jointly defined by all authors prior to creating a narrative structure. After having separately defined all of the themes for the three rounds of interviews, during the sixth and final phase of the thematic analysis, the analyses of each round of interviews were combined into one narrative in close consideration with all authors. The jointly executed analysis ultimately resulted in a scholarly report complemented by vivid and compelling quotations from the support staff.

## 3. Results

Four overarching themes emerged from the data: (1) balancing between one’s professional and personal life; (2) vaccination as both a stress reducer and a source of agitation; (3) service users: vulnerable versus resilient; and (4) contact with colleagues and service users.

### 3.1. Theme 1: Balancing between One’s Professional and Personal Life

During the initial phase of the COVID-19 pandemic, support staff were forced to maintain contact with and continue to support service users from home by engaging in online communication, such as videocalls, phone calls, and text messages. In conjunction with this, other preventive measures such as the closure of schools and other public spaces like nurseries meant that the support staff, who mostly had school-age children, had to also reorganize their own family situations. As a result of this novel reality, the support staff reported struggling to establish a new work-life balance, which was, according to them, especially challenging when they experienced stressful situations at work; for example, when they had an overly full caseload. In addition, the support staff described how they struggled to deal with their responsibility to continue to support their service users, and that they felt they had to compensate for not being able to make physical house visits. For example, some support staff began to leave their work phone on after working hours during this period so that their service users could reach out to them when they needed support.

As the pandemic continued for several months, during the second round of interviews, alongside struggling to maintain a work-life balance, the support staff also reported struggling to both sustain a positive mindset about the future and remain positive and realistic towards their service users. As one support staff member put it:

“In the first wave, I was not that scared, but during the second wave I felt down and struggled to remain optimistic about the future. I noticed that during this time, I found it hard to stay positive towards service users, giving them hope and showing them what we can still do or can appreciate. It takes a toll on us [support staff] to have the right conversation with all the different service users, showing them what the current reality is and how to work with the situation and give them a sense of relief and perspective.”

(Participant 7)

In addition, the support staff reported that they struggled to find a balance between their own wellbeing and the wellbeing of their service users. For instance, given that most service users were exhausted due to the pandemic, the support staff had to constantly comfort them and provide mental support, while, simultaneously, looking after their own personal wellbeing. The support staff expressed how they sometimes felt overwhelmed in these situations, and that they did not always know how best to support their service users.

At the time of the second and third interviews, at which point the support staff were able to conduct some house visits again, the outreach support staff described having more autonomy over complying with preventive measures in comparison to support staff working in residential care facilities who must adhere to the measures of their facility. However, this greater autonomy resulted in mixed feelings. On the one hand, support staff stated that certain preventive measures, such as wearing a facemask when visiting a service user, sometimes resulted in service users feeling distressed and uncomfortable, which, in turn, led to them not being welcome in their houses if they were wearing a facemask. However, on the other hand, not knowing either who their service users had been in contact with or whether they were being truthful about experiencing any coronavirus-related symptoms led some professionals to feel stressed, anxious, or cautious. In the words of one support staff member:

“I was getting used to working from home, but since I can make limited house visits, I have to contemplate daily about the possible risks of infection. I have to think: “oh he is now there, I was just there, etc., constantly weighing up the risks and never knowing if you did the right thing or not. You can only find out in the end whether you did it right and this is starting to bother me. I feel myself becoming insecure at times because I do not want to put anyone’s health at risk, but I also realize that some visits really must take place. I am really done with this constant thought process.”

(Participant 6)

Although the support staff indicated that they discussed these mixed feelings with their team manager and/or colleagues, in order to weigh up the respective risks and benefits, ultimately, the decision over whether to adhere to the preventive measures in specific cases invariably had to be made by the support staff themselves.

### 3.2. Theme 2: Vaccination as Both a Stress Reducer and a Source of Agitation

During the first round of interviews, several support staff expressed fear over the fact that they were at a higher risk of infection from COVID-19 due to their work, and, as such, that they longed to be vaccinated. During the second round of interviews, which took place at a point in which support staff were invited to receive their first COVID-19 vaccination, most of the support staff expressed feeling more hopeful in light of the progress of the vaccination program, although they were also relatively cautious due to prior obstacles with the vaccination program. Still, most support staff stated feeling confident about their decision to get vaccinated, as it would reduce the risk of infection when making house visits, and thus would make them feel more at ease when being around service users:

“I will definitely get vaccinated, because I feel like it will make me less hazardous, at least, I assume that [the vaccine] works properly and does not have serious side effects, that’s what they communicate now. Doing so will prevent me from getting infected by my service users, which may make me feel more relaxed. And I can’t bring [the virus] to the next [service user].”

(Participant 1)

However, during the second interview round, some of the support staff also reported experiencing doubts over deciding whether they wanted to get vaccinated or not. Searching through different information sources, media reports, and the opinions of their social network caused more distress and led to greater doubt. Although some of the support staff felt comfortable discussing their doubts and indecision with their colleagues, others felt either insecure about sharing this with their colleagues or pressured into forming an opinion, as others already appeared to have formed strong opinions about the vaccination program.

Support staff continued to report various concerns over the ongoing vaccination program during the third round of interviews. Due to the nature of their work, support staff were one of the first to get vaccinated in the Dutch vaccination program, which led some support staff to experience doubt and anxiety over having received a vaccine with possible side-effects that were still unknown due to their rapid development.

Increased media reporting about both the side-effects and ineffectiveness of the vaccine that most support staff were vaccinated with, resulted in a nagging feeling that they had received an inferior vaccine.

### 3.3. Theme 3: Service Users: Vulnerable versus Resilient

During the first lockdown, most support staff worried about the wellbeing of their service users. They observed how service users were struggling with both the complicated new reality posed by the COVID-19 pandemic and how to make sense of the situation. Moreover, the support staff expressed fear over the fact that the preventive measures might lead service users to feel depressed or lonely. According to the support staff, these fears were grounded in their pre-pandemic experiences of how service users found it difficult to both reach out for help and organize activities by themselves. During the second round of interviews, at which point the pandemic and its preventive measures had been in place for several months, the notion of the vulnerability of service users came to the fore even more:

“I notice that service users start to lose hope and realize that things may never be the same, especially because there is barely any progress now. This fear is starting to show with everybody, but especially with our service users. Service users have shown more signs of depression and loneliness recently.”

(Participant 3)

During the third round of interviews, which took place one year into the pandemic, support staff noted that some service users were “sick and tired” of the preventive measures and had started to behave more carelessly, for example, attending large social gatherings and inviting multiple visitors a day to their homes, which went against the governmental recommendations at that time. Given that many service users regarded the vaccine as the ultimate solution against COVID-19, the support staff indicated that non-compliance with the preventive measures increased from the very moment that service users received their first vaccine. According to support staff, this derived from the fact that service users assumed that they could no longer get infected or spread the virus after being vaccinated, which, in turn, led them to believe that they could return to their pre-pandemic behavior. Consequently, in order to prevent infection and the spread of the virus, the rationale behind the remaining preventive measures after getting vaccinated, such as avoiding shaking hands and limiting the number of visitors to your home, had to be repeatedly explained to service users, which some support staff found tiring.

Interestingly, whereas some support staff emphasized the vulnerability of service users, others expressed surprise over the resilience and independence that they had observed among some service users. This observation recurred throughout all three rounds of interviews, with support staff noting how well service users had coped throughout the different phases of the pandemic. This made support staff realize that the resilience and independence of service users may have been stronger prior to the pandemic than they thought:

“Because of the lockdown I noticed how service users sometimes need me less than I think and that is a positive realization (…) One service user started sorting out his mail by himself and called the sender of the mail if they had questions about the letters. Service users started to take on these tasks themselves because I was not there, calling for them, as I usually do.”

(Participant 1)

### 3.4. Theme 4: Contact with Colleagues and Service Users

Given that support staff were unable to visit their office for a long period of time due to the preventive measures, they came to realize how important in-person contact with colleagues was for them. During all three rounds of interviews, support staff stated that they valued discussing complex cases that required a second opinion with colleagues, especially during the pandemic:

“We rarely see each other these days because you always work solo, you’re always on the road. Because we have rather complex cases, we usually work together on different cases. This is great because we can discuss the service users or the events that occur, especially during this period….”

(Participant 3)

In addition, support staff valued their colleagues as persons who understood their frustrations, doubts, or questions. They described the value they placed on how their team tried to emotionally support each other during the ongoing pandemic and take over cases in the event of illness. In some cases, care organizations arranged online coffee dates for the team to help staff connect during the pandemic, which was appreciated by most of the support staff. As the pandemic continued, support staff described missing the connections with their colleagues even more. Prior to the pandemic, support staff stated that connecting with colleagues was a frequent occurrence in the office where they worked in between appointments. This was no longer possible once the pandemic began and the preventive measures came into effect, and support staff had to instead proactively reach out to their colleagues. During all rounds of interviews, support staff reported missing spontaneous forms of contact and indicated that the absence of this made their work feel even more individualistic and like everyone was struggling on their own island. Therefore, COVID-19 made the support staff cognizant of how important it is to have frequent contact with one another and that they should be more proactive in initiating it.

Next to mentioning the importance of in-person contact with colleagues, support staff also stressed the importance of in-person contact with service users. That is, faced with a novel reality in which support staff were forced to engage with service users using (video) calls, throughout the different rounds of interviews, support staff stated their preference for making house visits rather than engaging in (video) calls. This was because house visits enabled them to form a better understanding of their service users’ wellbeing. In addition, support staff underscored the importance of having service users’ full attention when having difficult conversations, which is easier to accomplish during house visits than it is during (video) calls. Some support staff even expressed that being forced to work remotely made them feel like they were doing maintenance work rather than working on a specific set of goals together with service users. Interestingly, despite their preference for house visits, during the third round of interviews, when support staff had around one year of experience of working remotely, support staff stated that they did not aspire to go back to the pre-pandemic situation, where their days entirely consisted of home visits. Most support staff considered (video) calls to be a very time-efficient way of supporting service users without compromising on the quality of support. This was especially the case with respect to attending service users’ appointments with other organizations that were held online, such as a meeting with a job coach. Moreover, support staff came to realize that some specific service users could better be supported remotely. For example, by making less house visits to specific service users, these service users could be supported more appropriately, thus strengthening their independency:

“I noticed throughout this process that you can direct some service users better using (video) calls. You can explain things to them and then they must do it themselves. I believe the difference here is that my hands literarily are not helping around the house. This makes me more aware about supporting service users without the use of hands. I notice the same realization with other colleagues. It will probably not work for all service users, but I really believe they have become more independent.”

(Participant 1)

Support staff also reported having gained a lot of time by not having to commute to the office and service users’ homes, which, in turn, enabled them to spend more time supporting each service user. Support staff indicated that they preferred to spend this extra time in a flexible manner, so that they could support service users more easily on demand.

“I used to have a really tight schedule, but now I have time when someone really needs me. This approach of catering to their needs allows me to support more clients in a shorter amount of time.”

(Participant 1)

Finally, throughout the interviews, support staff emphasized the importance of service users having sufficient contact moments, in order to receive adequate support and attention, especially during times of crises. Support staff reflected on their responsibilities in meeting those needs. For instance, as some support staff stated that service users struggled to make social contact themselves, they reiterated that it was their responsibility to organize daily activities and social contact for their service users.

## 4. Discussion

This study reported the experiences of outreach support staff supporting people with mild intellectual disabilities who were independently living in the community throughout different phases of the COVID-19 pandemic (March 2020–May 2021) in the Netherlands. Four overarching themes emerged: (1) balancing between one’s professional and personal life; (2) vaccination as both a stress reducer and a source of agitation; (3) service users: vulnerable versus resilient; and (4) contact with colleagues and service users. These themes provided valuable insights into the experiences of outreach support staff over time related to the different phases of the COVID-19 pandemic, during which various preventive measures were in place.

First, in the present study, outreach support staff explained the enduring impact that the COVID-19 pandemic and its preventive measures had upon their professional and personal lives. Although participants reported similar experiences to those expressed by support staff working within residential care facilities during the first phase of the COVID-19 pandemic (e.g., being fearful of getting infected [4]), they also discussed experiences that were specific to their job as outreach support staff. For instance, outreach support staff had greater autonomy over complying with the preventive measures when conducting house visits than their colleagues in residential care facilities. This autonomy often resulted in mixed feelings for the support staff, such as the struggle to outweigh the interests of the service users with their own interests. Being able to discuss these mixed feelings with colleagues was deemed to be imperative to limit the stress reported by support staff as much as possible, as caring for service users during the pandemic in itself already increased the risk of mental health problems for support staff [6,8,14,15]. In particular, McMahon et al. [8] reported that support staff that worked more independently during the COVID-19 pandemic reported higher levels of burn-out, anxiety, and depression. Providing proper support to outreach support staff during the pandemic is therefore of utmost importance; for example, by initiating peer support groups, which have proven to be effective to emotionally support frontline clinical workers during the COVID-19 pandemic [16]. 

Second, outreach support staff reported mixed attitudes regarding getting vaccinated. In general, support staff were in favor of getting vaccinated. However, being one of the first in line to get vaccinated also raised concerns, including concerns about the vaccines’ side effects and its fast development. These findings are in line with Iadarola et al. [17], concluding that fear of side effects, the notion of the vaccine being ‘too new’, and not wanting to be an ‘experiment’ for the vaccine were main attitudes that inspired vaccine hesitancy. Similar attitudes were reported by Lunsky et al. [18], who explored beliefs regarding COVID-19 vaccines among workers in the intellectual disability sector. These attitudes are echoed in research in other sectors, including elderly care (e.g., [19]) and hospital care (e.g., [20]). Further research on the experiences and beliefs of support staff regarding vaccines may provide relevant information for future booster campaigns for COVID-19 or large-scale vaccination programs in which support staff may once again be one of the first in line to get vaccinated.

Third, the different stages of the COVID-19 pandemic and the associated preventive measures challenged support staff to reflect on their attitudes regarding their service users and on the importance and form of contact with them. Over the course of the pandemic, support staff were able to explore their preferences towards both remote and in-person support. Although remote support was sometimes experienced as useful, efficient, and effective, which is in line with previous research (e.g., [21,22]), overall, support staff preferred meeting (new) service users in-person, as they felt this gave them a better impression of their wellbeing and living situation. In other specific cases, however, support staff experienced how service users could benefit from remote support. That is, some support staff experienced that service users demonstrated that they were more capable of doing things by themselves and that they acted more self-determinedly compared to the pre-pandemic period. These specific cases evoke the awareness of supporting service users more from an autonomy supportive approach, in which the environment creates opportunities for people to make their own decisions and to take control of their own lives (i.e., being self-determined; [23]). This is in line with the United Nations Convention on the Rights of Persons with Disabilities (UNCRPD; [24]), which emphasized that people with intellectual disabilities should have more opportunities for being self-determined. This is important, as self-determination is an essential dimension of quality of life [25] and has been linked to other positive outcomes for people with intellectual disabilities over the past decades (e.g., [26,27,28,29]). Future research is needed to explore whether the awareness of supporting service users more from an autonomy supportive approach remains in the post-pandemic era and how this influences the way support staff provide support to service users in the (near) future. Moreover, future research is needed to explore in what instances remote support is preferable for all stakeholders, including people with intellectual disabilities, both for themselves and their relatives. It is relevant is this respect to note that remote support may offer opportunities to support people with mild intellectual disability in their autonomy and self-determination [30]. Nevertheless, scientific research on this topic is in its early stage, and further high-quality research is needed [22].

The results of the present study should be interpreted in the light of some limitations. First, this study had a small sample size, thus preventing the possibility of generalizing the findings. The participants were purposefully selected by managers of the three participating organizations; all met the inclusion criteria, yet it is unclear on what specific grounds managers invited support staff to take part in this study. Although additional participants may have resulted in alternative themes or subthemes, the findings of this study nevertheless remain relevant and informative in that they document the experiences of outreach support staff over time related to different stages of the COVID-19 pandemic, which, to the best of our knowledge, has hitherto not been reported. Second, given the persistence of the COVID-19 pandemic, the study thus only encompasses part of the pandemic. However, by providing insights into the experiences of support staff during different phases of the pandemic, during which various preventive measures were in place that influenced their work, we were able to provide valuable insights into the enduring impact of the pandemic upon outreach support staff.

## 5. Conclusions

To conclude, the present study provided valuable insights into the experiences of outreach support staff working with people with mild intellectual disabilities during different stages of the COVID-19 pandemic, as well as the enduring impact of the pandemic upon them. Specifically, the study showed that outreach support staff experienced struggles during the COVID-19 pandemic that were specific to the autonomous and individual nature of their occupation. In addition, the study provided insights into the experiences and beliefs of support staff regarding vaccines and being first in line to get vaccinated. Finally, the present study provided valuable insights in how being forced to remotely support service users evoked the awareness of supporting service users more from an autonomy supportive perspective.

## Data Availability

The research data cannot be shared.
